# Supplementary figures and images for: Ustilago maydis telomere protein Pot1 harbors an extra N-terminal OB fold and regulates homology-directed DNA repair factors in a dichotomous and context-dependent manner
Source: PLoS Genet. 2022 May 19;18(5):e1010182. doi: 10.1371/journal.pgen.1010182 (PMC9119445; doi:10.1371/journal.pgen.1010182)

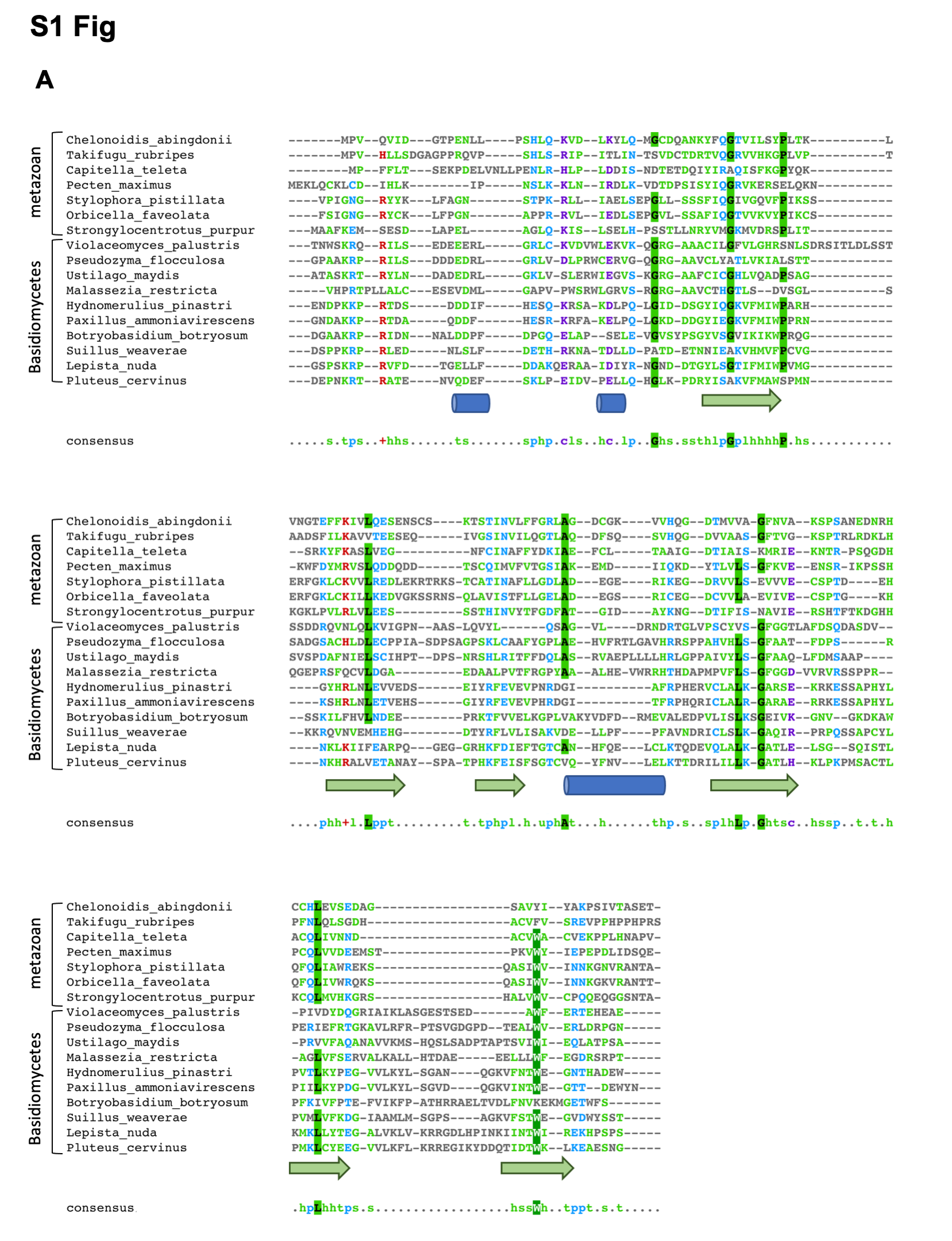

Supplement: S1 Fig — A. Multiple sequence alignment of the putative OB-N domains from metazoans and basidiomycetes. The alignment and secondary structure predictions were performed using PROMAL3D and displayed using MView. Amino acids were colored by groups. B. Multiple sequence alignment of the OB3 domains of Pot1 homologs from Basidiomycota, Chytridiomycota, and Zoopagomycota. C. Multiple sequence alignment between the OB3 domains of Pot1 homologs from Basidiomycota and Ascomycota. The putative Zinc-binding residues in the basidiomycete OB3 domains are highlighted. (TIF) [file pgen.1010182.s001.tif]

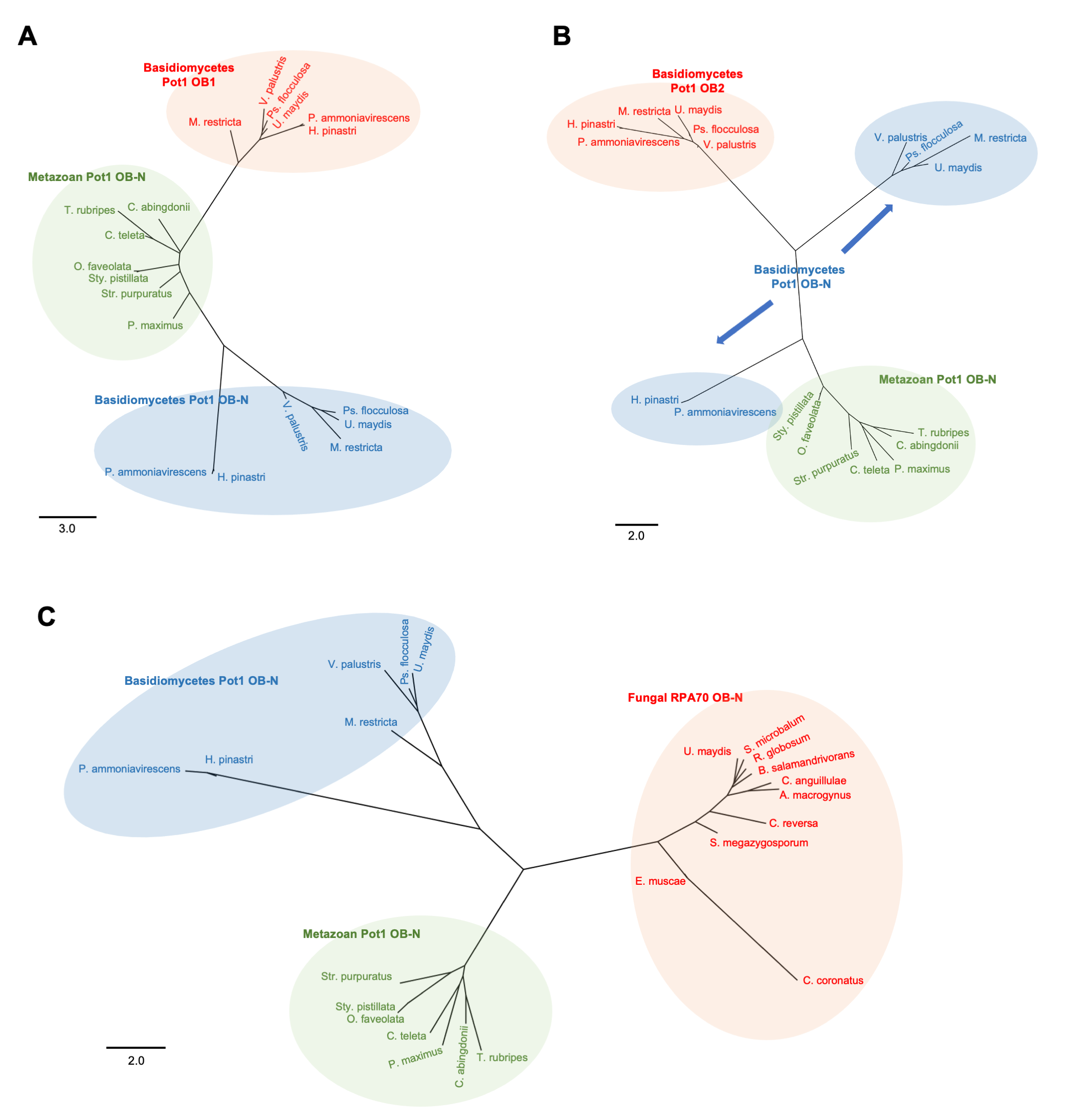

Supplement: S2 Fig — A. To analyze the relatedness of basidiomycete Pot1 OB1 to the OB-N domains from basidiomycete and metazoan Pot1, we generated a multiple sequence alignment between these three groups of OB folds using T-Coffee. The Phylip alignment was used to infer phylogeny via FastME 2.0 (http://www.atgc-montpellier.fr/fastme/) [54] and the result was plotted using FigTree [55]. B. The same analysis was performed for basidiomycete Pot1 OB2 vis-à-vis basidiomycete and metazoan Pot1 OB-N domains. C. The same analysis was performed for fungal RPA1 OB-N vis-à-vis basidiomycete and metazoan Pot1 OB-N domains. (TIF) [file pgen.1010182.s002.tif]

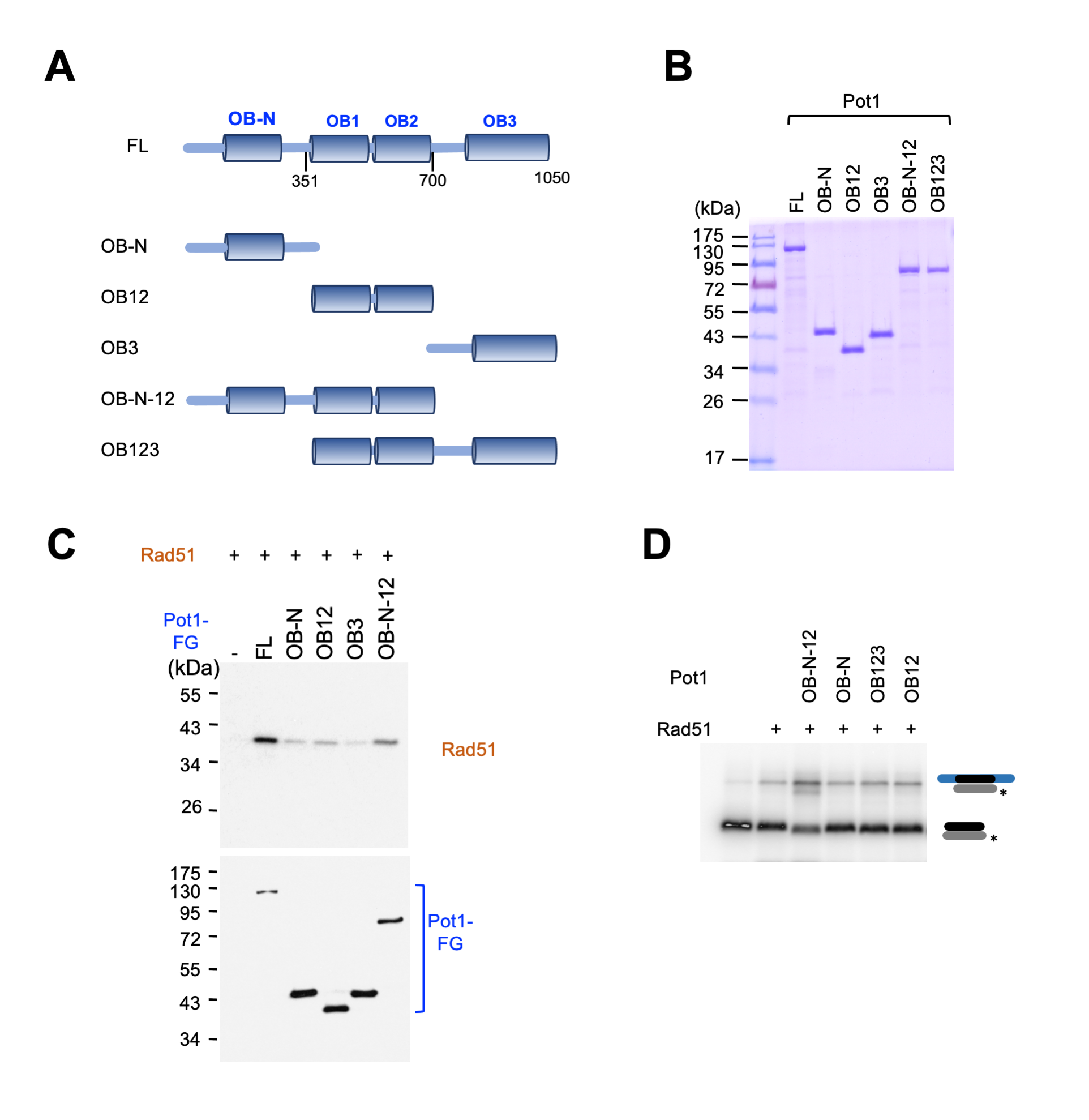

Supplement: S3 Fig — A. The domain structure of Pot1 and the truncation variants analyzed in this study are shown. B. Affinity purified Pot1 proteins were analyzed by SDS-PAGE and Coomassie staining. C. Purified Rad51 was subjected to pull down analysis using FLAG-tagged Pot1 and truncation derivatives. The eluates were analyzed via Western using anti-Rad51 (for Rad51) and anti-FLAG (for Pot1) antibodies. D. The effects of Pot1 truncations on the strand exchange activity of Rad51 were analyzed using oligonucleotide substrates. (TIF) [file pgen.1010182.s003.tif]

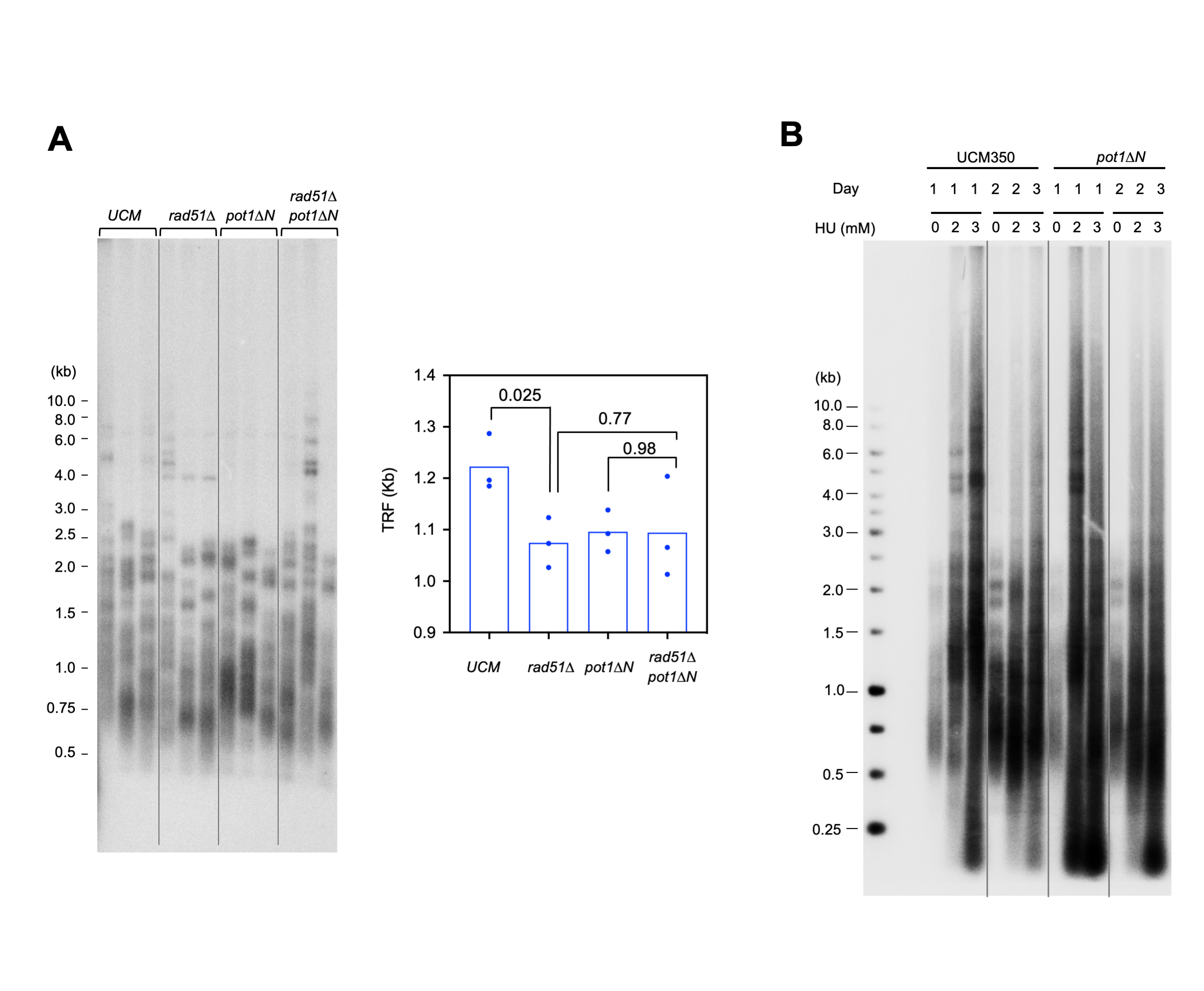

Supplement: S4 Fig — A. Chromosomal DNAs from three independently propagated cultures of each strain were isolated after ~100 generations of growth (4 streaks) and subjected to telomere restriction fragment analysis. The blot is displayed on the left and the average TRF lengths plotted on the right. B. The UCM350 and pot1ΔN strains were grown in YPD liquid cultures with the indicated concentrations of hydroxyurea and for the specified numbers of days. Chromosomal DNAs were isolated and subjected to telomere restriction fragment analysis. (TIF) [file pgen.1010182.s004.tif]

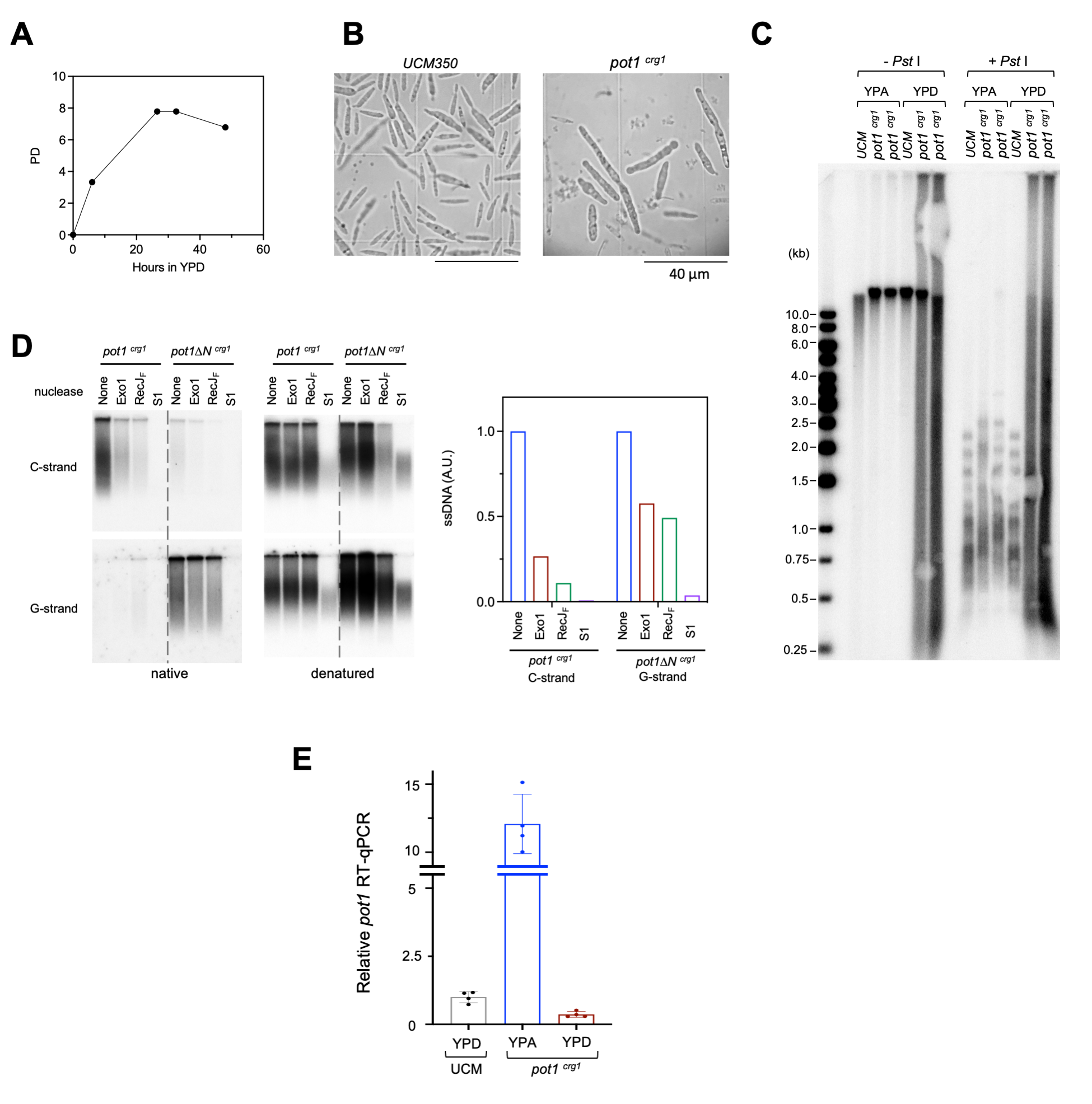

Supplement: S5 Fig — A. A pot1crg1 liquid culture grown in YPA was harvested, washed 3 times with water, and resuspend in YPD to a starting OD600 of 0.1. The population doubling of the culture was then monitored over a period of 48 hours. B. The UCM350 and pot1crg1 strains were first grown in YPA, and then switched to YPD medium. After another 24 hours of growth, the cells were examined under the microscope. C. Chromosomal DNAs from the indicated strains grown in either YPA or YPD were isolated and subjected to Southern analysis with a telomere repeat probe (TR82) with or without prior PstI digestion. D. DNAs from pot1crg1 and pot1ΔNcrg1 grown in YPD were treated with the indicated nucleases and then subjected to in-gel hybridization analysis of G- and C-strand ssDNA. The signals were normalized against the untreated sample, and then plotted on the right. E. The pot1 RNA levels from the indicated strains grown in the specified media were analyzed by RT-qPCR. The results were normalized against the signal for UCM grown in YPD, and then plotted. (TIF) [file pgen.1010182.s005.tif]

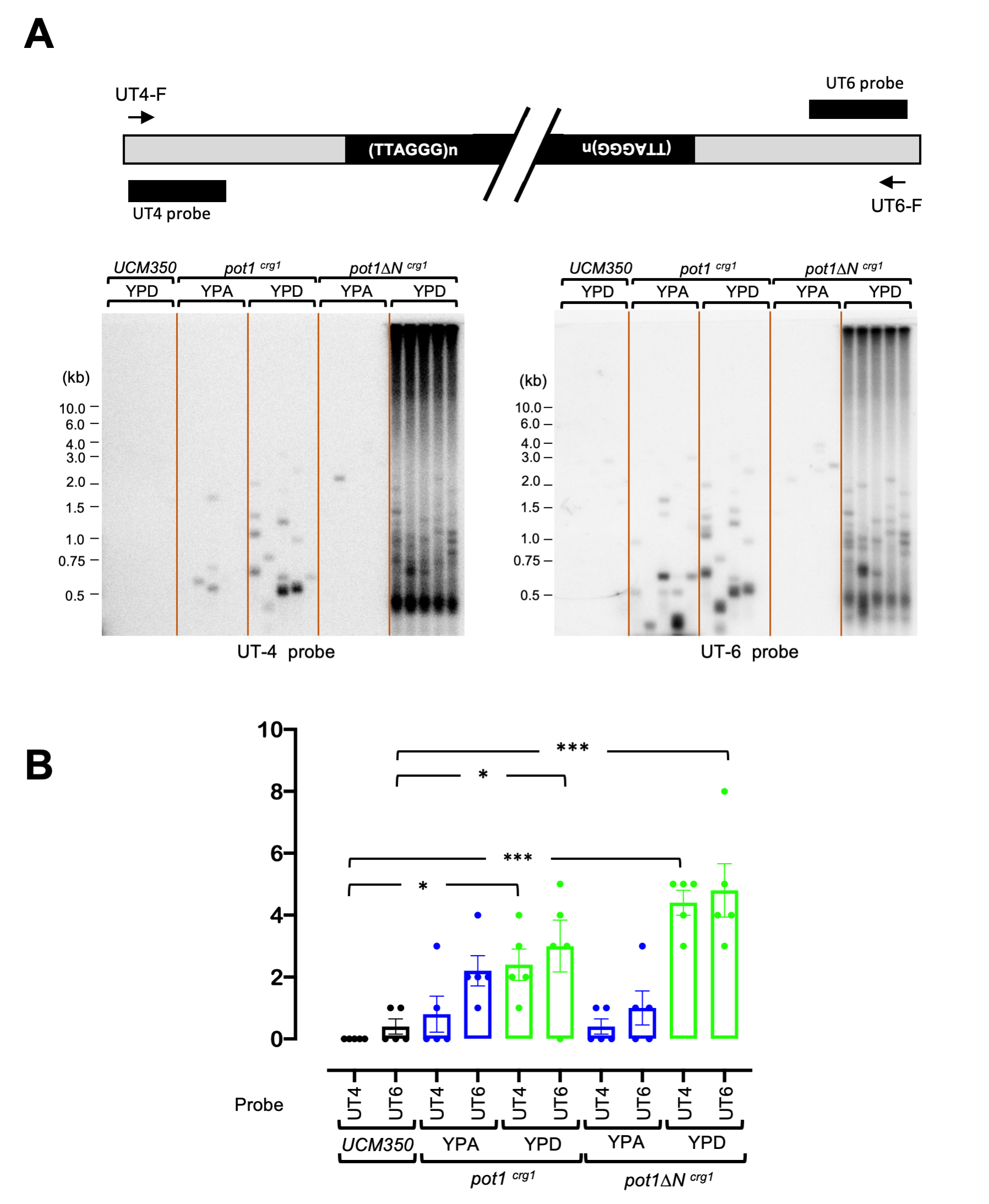

Supplement: S6 Fig — A. (Top) Schematic diagram of the primers and probes used to detect fusions between UT4- and UT6-containing telomeres. (Bottom) Chromosomal DNAs from the indicated strains grown in either YPA or YPD were subjected to PCR-based fusion detection. Five independent PCR reactions per DNA sample were performed; the products were separated by electrophoresis and subjected to Southern analysis using sequentially a UT4 and a UT6 probe. B. The number of fusion fragments were determined and plotted. Statistical significance was calculated using Students’ test (*, <0.05; **, <0.01; ***, <0.001). (TIF) [file pgen.1010182.s006.tif]

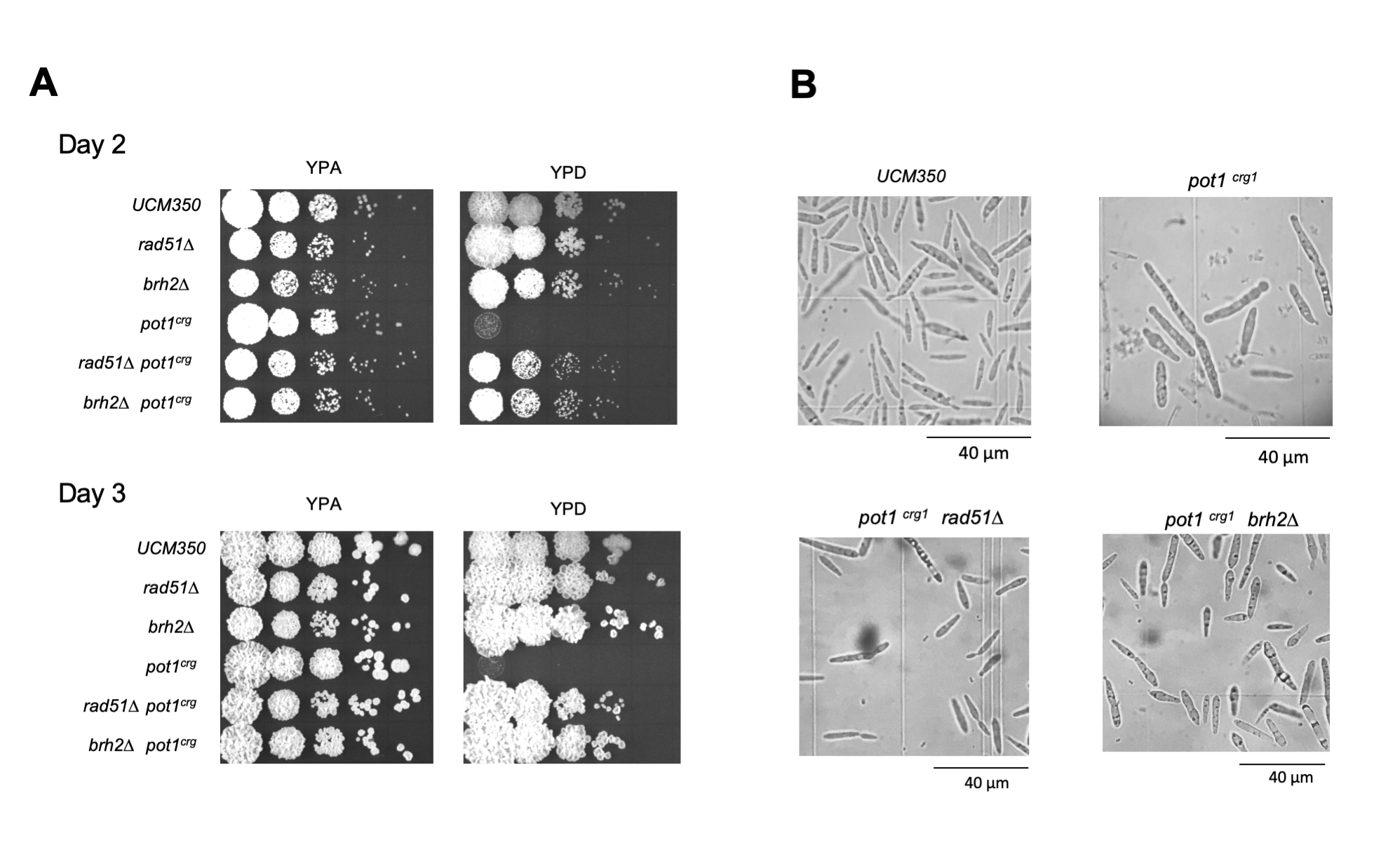

Supplement: S7 Fig — A. Serial dilutions of the indicated strains were spotted on YPA and YPD plates. The growth of the strains after 2 and 3 days of incubation were imaged. B. The morphologies of the indicated strains after 24 hours of growth in YPD were examined. (TIF) [file pgen.1010182.s007.tif]

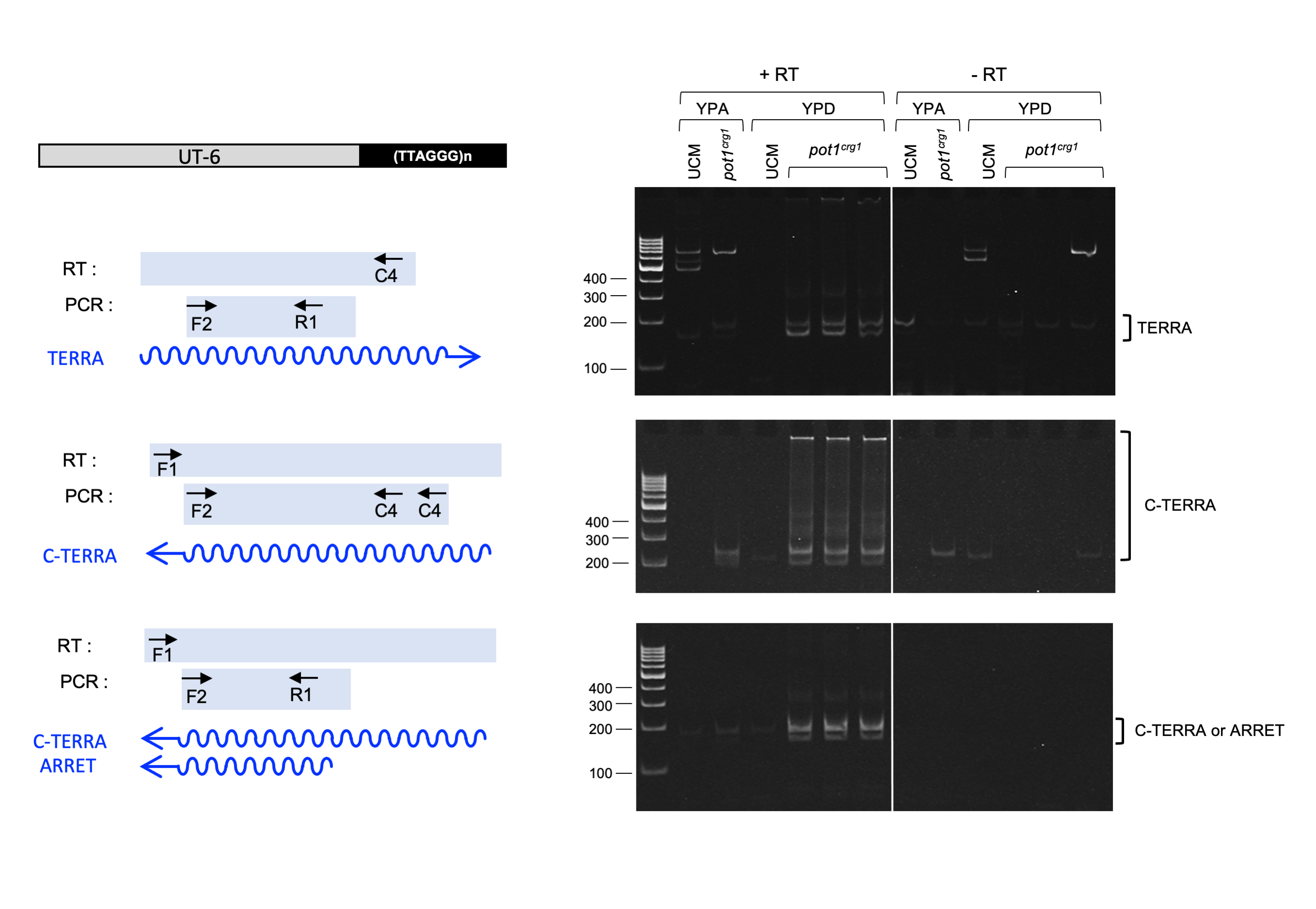

Supplement: S8 Fig — (Left) Schematic illustrations of the structure of UT6 telomeres and the primers used in the RT and PCR reactions designed to detect telomere repeat RNAs. Note that ARRET was previously defined as C-strand RNA comprised of subtelomere sequences only. (Right) RNA samples from the indicated strains grown in YPA or YPD were subjected to RT-PCR using the primers indicated to the left of each panel. Both the F1/R2 and F2/R1 PCR reactions are expected to generate ~200 bp products. The multiple bands detected are likely due to minor insertions/deletions in different copies of UT6 elements [22]. (TIF) [file pgen.1010182.s008.tif]

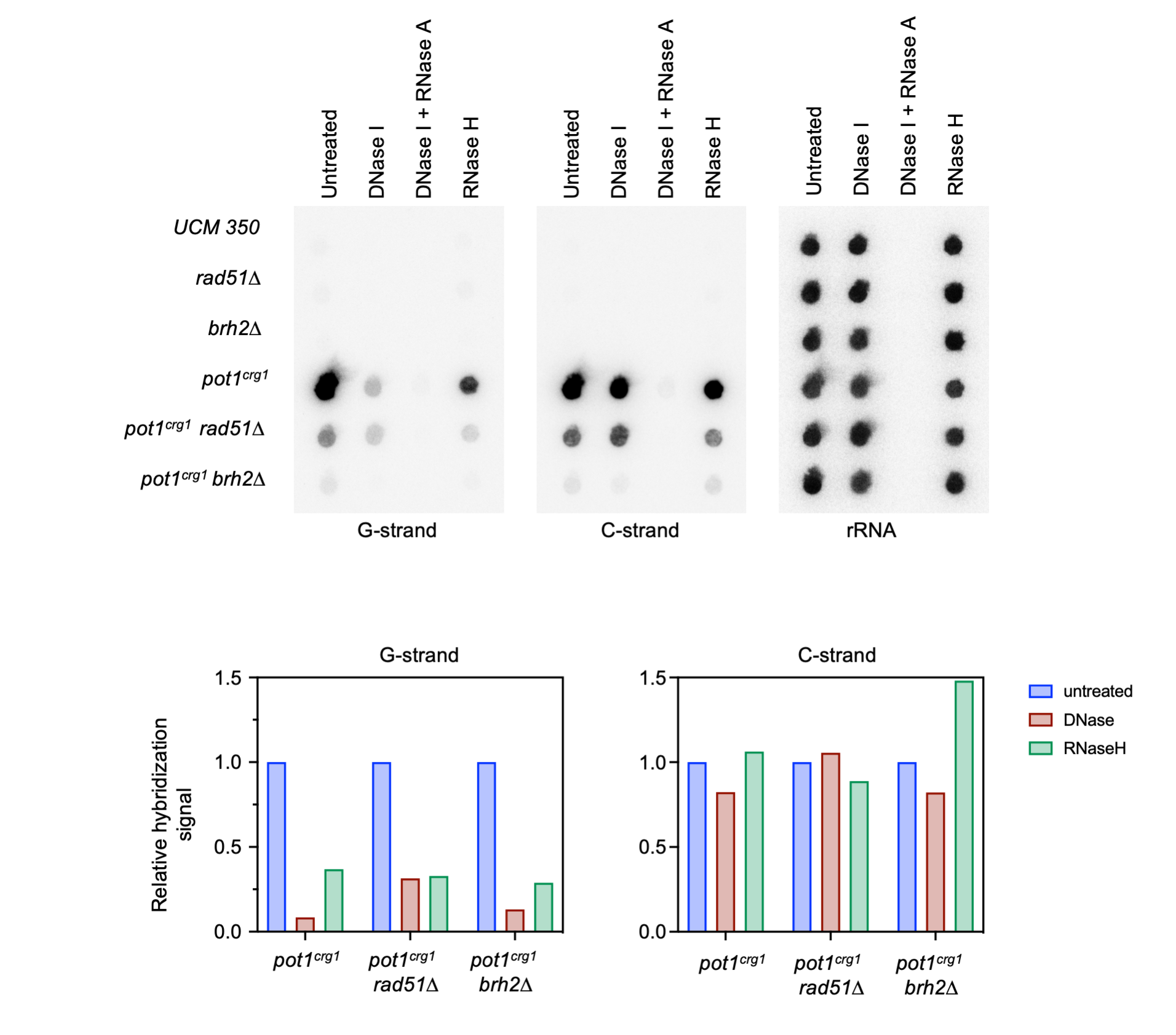

Supplement: S9 Fig — (Top) RNAs from the indicated strains grown in YPD were treated with the indicated nucleases and then subjected to dot blot analyses using sequentially probes for detecting G-strand RNA, C-strand RNA, and 26S rRNA. (Bottom) The ratios of G-strand and C-strand signals to rRNA signals for the untreated, DNase-treated and RNase H-treated samples were calculated. Each ratio was normalized against the untreated sample for the particular strain and plotted. (TIF) [file pgen.1010182.s009.tif]

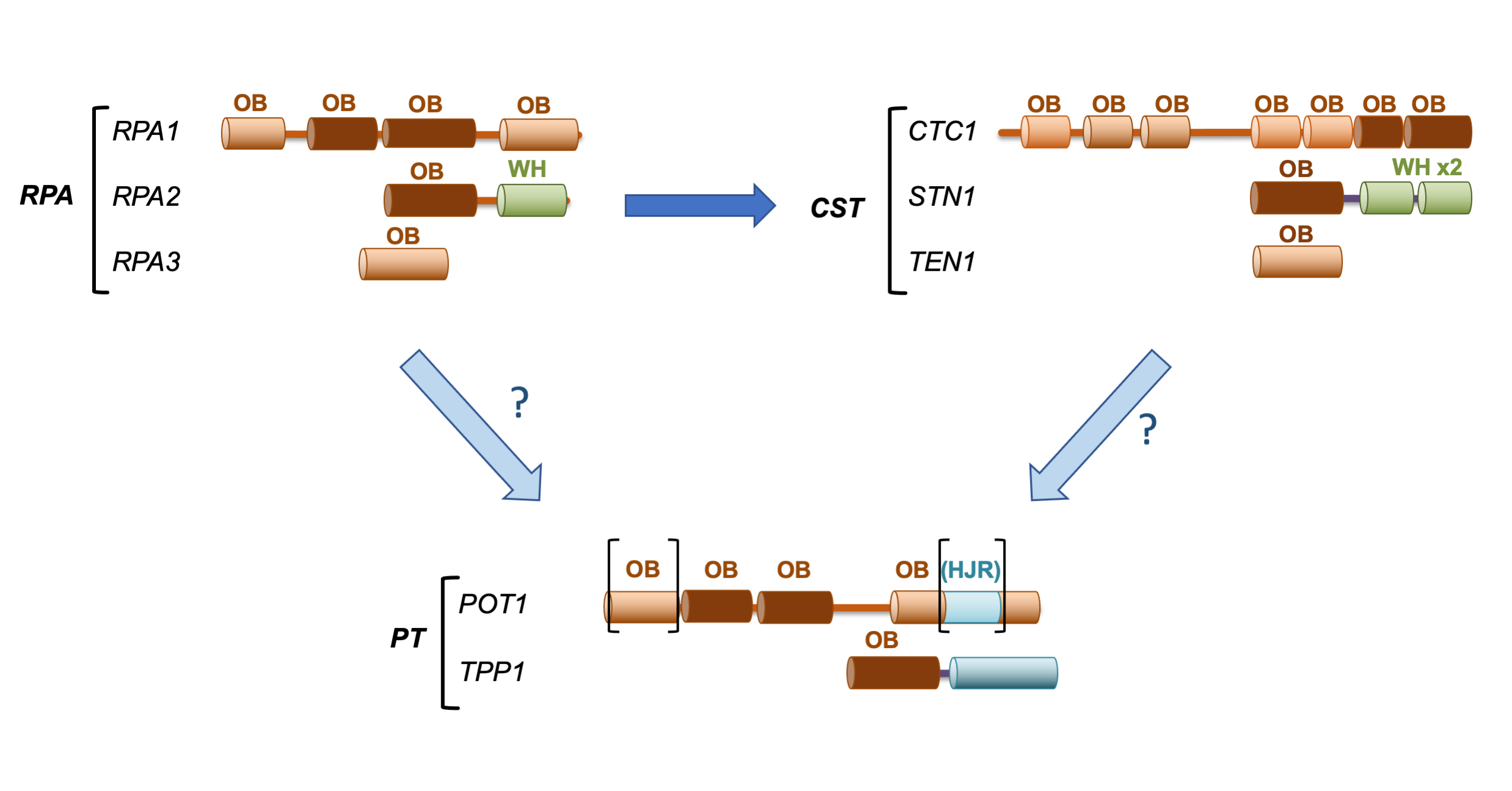

Supplement: S10 Fig — The domain structures of RPA, CST and PT subunits are illustrated schematically. The designations for the domains are as follows: OB, oligosaccharide/oligonucleotide-binding; WH, winged-helix; HRJ, Holliday junction resolvase. The OB folds implicated in ssDNA-binding are shaded in dark brown. Available structural and functional evidence strongly suggests that RPA and CST share a common ancestry. Whether PT is derived from the same primordial ssDNA-binding complex is less clear. The demonstration of a 4-OB architecture for Pot1 orthologs in fungi and metazoan suggests that Pot1 and RPA1 have similar domain organizations and may indeed be evolutionarily related. (TIF) [file pgen.1010182.s010.tif]
