# Supplementary material for: Ustilago maydis telomere protein Pot1 harbors an extra N-terminal OB fold and regulates homology-directed DNA repair factors in a dichotomous and context-dependent manner
Source: PLoS Genet. 2022 May 19;18(5):e1010182. doi: 10.1371/journal.pgen.1010182 (PMC9119445; doi:10.1371/journal.pgen.1010182)
Supplement: S1 Table — (DOCX) [file pgen.1010182.s011.docx]

**S1 Table. *U. maydis* strains used in this study**

| **Alias (Haploids)** | ***Relevant Genotype*** | **Reference** |
| --- | --- | --- |
| UCM350 ^a^ | wild type | Kojic et al., 2002 [1] |
| USZ104 ^ab^ | *pot1∆N* | This work |
| USZ105 ^ac^ | *pot1^crg^* | This work |
| USZ106 ^ad^ | *pot1∆N^crg^* | This work |
| USZ107 ^ae^ | *rad51∆* | This work |
| USZ108 ^ace^ | *pot1^crg^ rad51∆* | This work |
| USZ109 ^af^ | *brh2∆* | This work |
| USZ109 ^acf^ | *pot1^crg^ brh2∆* | This work |

^a^ The genotype of UCM350 *is nar1-6 pan1-1 a1b1*. *nar*, *pan*, and *ab* indicate inability to reduce nitrate, auxotrophic requirement for pantothenate, and mating type loci, respectively.

^b^ *pot1* was replaced with an N-terminally truncated allele; a *cbx* cassette expressing the carboxin resistance gene (Cbx^R^) was inserted upstream of the promoter.

^c^ *pot1* promoter was replaced by the *crg1* promoter

^d^ *pot1* promoter was replaced by the *crg1* promoter, and the first 1125 nt of the ORF was deleted.

^e^ *rad51* was disrupted by a *nat^R^*-containing cassette.

^f^ *brh2* was disrupted by a *nat^R^*-containing cassette.

**References**

1. KOJIC, M., KOSTRUB, C. F., BUCHMAN, A. R. & HOLLOMAN, W. K. 2002. BRCA2 homolog required for proficiency in DNA repair, recombination, and genome stability in Ustilago maydis. *Mol Cell,* 10**,** 683-91.
