# Supplementary material for: Ustilago maydis telomere protein Pot1 harbors an extra N-terminal OB fold and regulates homology-directed DNA repair factors in a dichotomous and context-dependent manner
Source: PLoS Genet. 2022 May 19;18(5):e1010182. doi: 10.1371/journal.pgen.1010182 (PMC9119445; doi:10.1371/journal.pgen.1010182)
Supplement: S2 Table — (DOCX) [file pgen.1010182.s012.docx]

**S2 Table. Oligos used in this study**

| **Name** | **Sequence 5’ to 3’** |
| --- | --- |
| **Protein Expression** |  |
| UmPot1-F-Bam | AAT GGATCC atg ccg cgc aag aga aaa ggt acc |
| UmPot1-R-FG-NotI | AAT GCGGCCGC cta CTT GTC ATC GTC ATC CTT GTA ATC caa tag atc gtg ttc gtc aga t |
| UmPot1-700-R-FG-NotI | AAT GCGGCCGC cta CTT GTC ATC GTC ATC CTT GTA ATC tcg atc ggc cag cgc ttc ttg |
| UmPot1-350-R-FG-NotI | AAT GCGGCCGC cta CTT GTC ATC GTC ATC CTT GTA ATC agc tct gga tgg att tgg tga a |
| UmPot1-351-F-Bam | AAT GGATCC ttg tcg agg caa gct tcc gct |
| UmPot1-701-F-Bam | AAT GGATCC ctc gag cga caa tcc agc ca |
|  |  |
| **Strain Construction and Genotyping** |  |
| UmPot1-F-Bam | AAT GGATCC ATG CCG CGC AAG AGA AAA GGT ACC |
| UmPot1-R-FG-NotI | AAT GCGGCCGC CTA CTT GTC ATC GTC ATC CTT GTA ATC CAA TAG ATC GTG TTC GTC AGA T |
| UmPot1-700R-FG-NotI | AAT GCGGCCGC CTA CTT GTC ATC GTC ATC CTT GTA ATC TCG ATC GGC CAG CGC TTC TTG |
| UmPot1-350R-FG-NotI | AAT GCGGCCGC CTA CTT GTC ATC GTC ATC CTT GTA ATC AGC TCT GGA TGG ATT TGG TGA A |
| UmPot1-351F-Bam | AAT GGATCC TTG TCG AGG CAA GCT TCC GCT |
| UmPot1-701F-Bam | AAT GGATCC CTC GAG CGA CAA TCC AGC CA |
|  |  |
| UmPot1(1)Nde | GAAATC CAT ATGCCGCGCAAGAGAAAA |
| UmPot1(700)Xba | ATT TCTAGA AGGAGGAGGCGAAAGCTG |
| UmPot1(-700)Xba | ATA TCTAGA TCGCAAATCGTGACGTGT |
| UmPot1(-1)Eco | ATT GAATTC GATGGATGTGATTCTGGA |
| UmPot1(1126)Nde | GAAATC CAT ATGGGCAACTGCCTCTAC |
| UmPot1(1825)Xba | ATT TCTAGA CGGTGTGGTCGGGATAG |
|  |  |
| Pot1-up-200 | TGACGTGTGACTGACATGC |
| Pot1-1100R (1117-1099) | GCTTGACGTTGGGCGCATT |
| Pot1-1900R (3920-3900) | GTTTTGGATGCGGATGATGTC |
| UmCrg_prom-200 | TGCAACATGAAGTTAGGTGTAGGC |
| Pot1-PCR-1576F | GCCAATTGGTTCTCCGAGCTTCAAG |
| Pot1-PCR-1898R | CCTGTCTTGAGGCCTTCTGCAATGG |
| Pot1-PCR-1801R | GACAGTCTGTGGGCAGATACCTGTC |
|  |  |
| **Strand exchange assays** |  |
| Strand_ex_70mer | AGTAGACTCAGCGAACTCACTGATCCAGTCTTAGCATCAGTCACGATACCTCGAGATACATACGGACGTA |
| Strand_ex_39mer_1 | TGATCCAGTCTTAGCATCAGTCACGATACCTCGAGATAC |
| Strand_ex_39mer_2 | GTATCTCGAGGTCTCGTGACTGATGCTAAGACTGGATCA |
|  |  |
| **PCR, hybridization, and EMSA assays** |  |
| TTAGGG_4_ (G4) | TTAGGG TTAGGG TTAGGG TTAGGG |
| CCCTAA_4_ (C4) | CCCTAA CCCTAA CCCTAA CCCTAA |
| TTAGGG_8_ (G8) | TTAGGG TTAGGG TTAGGG TTAGGG TTAGGG TTAGGG TTAGGG TTAGGG |
| CCCTAA_8_ (C8) | CCCTAA CCCTAA CCCTAA CCCTAA CCCTAA CCCTAA CCCTAA CCCTAA |
| UmrRNA_26S_121F | GCTTCGGACCATGCCTAAG |
| UmrRNA_26S_642R | CTTGGTCCGTGTTTCAAGACG |
|  |  |
| **RT-PCR and RT-qPCR** |  |
| UT6-TERRA-F1 | GGACGGCAGATATATATTGTGAGTGG |
| UT6-TERRA-F2 | GTGGCAACATTGGGTGAGC |
| UT6-TERRA-R1 | CCGTTGACACATTCAATCCCTC |
| UT6-TERRA-R2 | CTTCAAGCCCTGCAGCC |
| CCCTAA_4_ (C4) | CCCTAA CCCTAA CCCTAA CCCTAA |
|  |  |
| Pot1-PCR-3070R | acggatacgagtagacggcg |
| Pot1-PCR-2821F | gaatttgtcgagcgcaaatcgagg |
| Pot1-PCR-3020R | tctggatggagcaaacggttctg |
|  |  |
| **STELA and fusion assays** |  |
| UT4-F | tcgggcaacgttccatgtcg |
| UT4-subtel-R2375 | Ccctcgaaggcagtgcatac |
| UT6-F | ctactacacatcggttcaggc |
| UT6-subtel-R2400 | atgccaaagtggaaatcgtgcac |
| C Telorette 1 | GCTCCGTGCATCTGGCATCCCCTAAC |
| C Telorette 2 | GCTCCGTGCATCTGGCATCTAACCCT |
| C Telorette 3 | GCTCCGTGCATCTGGCATCCCTAACC |
| C Telorette 4 | GCTCCGTGCATCTGGCATCCTAACCC |
| C Telorette 5 | GCTCCGTGCATCTGGCATCAACCCTA |
| C Telorette 6 | GCTCCGTGCATCTGGCATCACCCTAA |
| Teltail | GCTCCGTGCATCTGGCATC |
