# Supplementary material for: Ustilago maydis telomere protein Pot1 harbors an extra N-terminal OB fold and regulates homology-directed DNA repair factors in a dichotomous and context-dependent manner
Source: PLoS Genet. 2022 May 19;18(5):e1010182. doi: 10.1371/journal.pgen.1010182 (PMC9119445; doi:10.1371/journal.pgen.1010182)
Supplement: S3 Table — (DOCX) [file pgen.1010182.s013.docx]

**S3 Table. Pot1 and RPA homologs utilized for *in silico* analysis in this study**

| **Classification** | **Organism** | **Protein ID** |
| --- | --- | --- |
| Basidiomycota Pot1 | *Ustilago maydis* | XP_011388186 |
|  | *Pseudozyma flocculosa* | SPO37959 |
|  | *Malassezia restricta* | XP_027482707 |
|  | *Lepista nuda* | KAF9469263 |
|  | *Suillus weaverae* | KAG2350098 |
|  | *Hydnomerulius pinastri* | KIJ69117 |
|  | *Paxillus ammoniavirescens* | KAF8844831 |
|  | *Pluteus cervinus* | TFK76258 |
|  | *Violaceomyces palustris* | PWN51691 |
|  | *Botryobasidium botryosum* | KDQ17907 |
| Ascomycota Pot1 | *Schizosaccharomyces japonicus* | XP_002173406 |
|  | *Zopfia rhizophila* | KAF2190160 |
|  | *Alternaria atra* | XP_043173166 |
|  | *Lasiodiplodia theobromae* | KAB2573190 |
|  | *Aspergillus sydowii* | XP_040708839 |
|  | *Leptosphaeria maculans* | XP_003840098 |
|  | *Xylona heveae* | XP_018192102 |
|  | *Saitoella complicata* | GAO49222 |
|  | *Botryosphaeria dothidea* | KAF4308573 |
|  | *Alectoria fallacina* | CAF9934687 |
|  | *Aspergillus niger* | GAQ40352 |
|  | *Elaphomyces granulatus* | OXV06789 |
|  | *Schizosaccharomyces octosporus* | XP_013016945 |
|  | *Schizosaccharomyces pombe* | NP_594453 |
| Chytridiomycota Pot1 | *Batrachochytrium dendrobatidis* | XP_006682830 |
|  | *Synchytrium endobioticum* | TPX41259 |
|  | *Neocallimastix JGI-2020a* | KAG4088494 |
|  | *Piromyces finnis* | ORX40370 |
| Zoopagomycota Pot1 | *Coemansia reversa* | PIA18514 |
|  | *Linderina pennispora* | XP_040739463 |
|  | *Smittium angustum* | PVZ97799 |
|  | | |
| Metazoan POT1 | *Chelonoidis abingdonii* | XP_032656845 |
|  | *Takifugu rubripes* | XP_011605395 |
|  | *Capitella teleta* | ELU00652 |
|  | *Pecten maximus* | XP_033744930 |
|  | *Stylophora pistillata* | XP_022798139 |
|  | *Orbicella faveolata* | XP_020608671 |
|  | *Strongylocentrotus purpuratus* | XP_030828861 |
|  | | |
| Fungal RPA1 | *Ustilago maydis* | XP_011388213 |
|  | *Conidiobolus coronatus* | KXN73660 |
|  | *Entomophthora muscae* | KAF7746839 |
|  | *Rhizoclosmatium globosum* | ORY52620 |
|  | *Catenaria anguillulae* | ORZ31153 |
|  | *Batrachochytrium salamandrivorans* | KAH9262341 |
|  | *Allomyces macrogynus* | KNE72602 |
|  | *Coemansia reversa* | PIA14594 |
|  | *Smittium megazygosporum* | PVV02755 |
|  | *Synchytrium microbalum* | XP_031027121 |
|  |  |  |
