# Supplementary material for: Ustilago maydis telomere protein Pot1 harbors an extra N-terminal OB fold and regulates homology-directed DNA repair factors in a dichotomous and context-dependent manner
Source: PLoS Genet. 2022 May 19;18(5):e1010182. doi: 10.1371/journal.pgen.1010182 (PMC9119445; doi:10.1371/journal.pgen.1010182)
Supplement: Attachment — Submitted filename: Critiques.pdf [file pgen.1010182.s015.pdf]

RE: PGENETICS-D-21-01605
Title: Ustilago maydis telomere protein Pot1 harbors an extra N-terminal OB fold and regulates homology-directed DNA repair factors in a dichotomous and context dependent manner

Authors: Syed Zahid, Sarah Aloe, Jeanette H. Sutherland, William K. Holloman, Neal F. Lue

Critiques

Zahid et al. reported the functions of *Ustilago maydis* Pot1 in telomere maintenance and protection in this manuscript. The authors found that *Ustilago* Pot1 has an extra OB fold at its N-terminus, interacts with Rad51 and, interestingly, stimulates Rad51's strand exchange activity. Using the arabinose-responsive crg1 promoter, the authors were able to overexpress POT1/pot1ΔN (when growing cells on YPA) or turn their expression off (when growing cells on YPD). The authors showed that Pot1 is essential for cell growth. Depletion of Pot1 leads to greater telomere length heterogeneity, an elevated telomeric C-circle level, increased amounts of single-stranded C and G-rich telomeric DNA, increased levels of both C and G strand TERRA, and more telomeric RNA:DNA hybrids. Many of these phenotypes are suppressed by rad51Δ and brh2Δ. The data support the conclusion that Pot1and Rad51 play critical roles in telomere maintenance and telomere recombination.

Concerns:

1. There is no estimation of crg1 driven POT1/pot1ΔN expression at the RNA or protein level when cells were grown on YPA and YPD compared to POT1 expression driven by its endogenous promoter. Some of the phenotypes, particularly for pol1ΔN growing on YPA, probably results from the overexpression of this mutant. In addition, if crg1 driven POT1/pot1ΔN expression can be depleted completely, the phenotypes for both POT1 and pot1ΔN cells grown on YPD is expected to be the same. The fact that they have very different phenotypes suggests that the depletion is not complete.
2. As a relevant point, POT1 OB1/2 and OB3 both can interact with RAD51, albeit the affinity appears to be lower for OB3. Still, this could contribute to the phenotypes of crg1 driven pot1ΔN grown on YPA. This is not discussed.
3. rad51Δ and brh2Δ suppressed the increased TERRA (both C and G strand) level in the pot1 mutant. This may be an effect on TERRA transcription, but it may also be due to increased TERRA degradation (or reduced TERRA stability). No evidence has been shown that the suppression is at the transcription level. Therefore, the statement on page 9 line 282 should be revised.
