# Supplementary material for: Ustilago maydis telomere protein Pot1 harbors an extra N-terminal OB fold and regulates homology-directed DNA repair factors in a dichotomous and context-dependent manner
Source: PLoS Genet. 2022 May 19;18(5):e1010182. doi: 10.1371/journal.pgen.1010182 (PMC9119445; doi:10.1371/journal.pgen.1010182)
Supplement: Attachment — Submitted filename: Response to Reviewers comments.pdf [file pgen.1010182.s016.pdf]

Response to Reviewers' comments

Reviewer #1:

This manuscript by Zahid et al. uses both biochemical and genetic approaches to examine the role of the telomeric protein Pot1 in the fungus U. maydis. The data provide an important step forward in understanding the role of this protein at telomeres in U. maydis, a model organism with arguably more telomeric similarities to metazoans than most other fungal model systems. Although most of the results reported bear some similarities to past results in mammalian or other systems, the manuscript should still be of quite broad interest in the telomere field. The manuscript is well written and the results presented are generally of high quality. Some specific criticisms are given below.

P2, l 47-48: This sentence should be altered a bit. I would not say that Rad51 is involved in completing telomere replication as this has not been shown directly here. Length regulation may be perturbed but this is not the same thing.
The sentence is modified to indicate that Rad51 is involved in "facilitate the maintenance of telomere DNA" (page 2, line 48).

P3, l 67-70: I suggest modifying the description of telomere erosion. Replication fork blockages by themselves do not shorten telomeres. Presumably, the subsequent processing is doing the actual shortening. Also, substantial erosion will occur via truncations from damage or recombination that are likely independent of replication fork blocks.
The sentence has been modified to incorporate these ideas (page 3, line 68-69).

It would be useful to mention in the text early on what the sequence and lengths of U. maydis telomeres are.
This is mentioned on page 4, line 96-97.

P5: Some additional information and perhaps analyses should be added regarding the discussion of the additional OB-fold (OB-N). One question is whether five predicted beta folds separated by an alpha helix is adequate as defining an OB-fold. The two predicted structures shown (Fig. 1C) seem rather different from one another and not obviously equivalent (to me at least) to many established beta barrel OB folds and the author's own evidence indicates that OB-N isn't involved in DNA binding. Also, where the predicted secondary structural features are should be clearly indicated in Fig. 1B (separately for each of the two species where a predicted structure is shown). Another matter is the question of the origin of the extra OB-fold. The authors favor the idea that it is an ancestral trait, conserved with the N-terminal OB-fold of metazoans. However, the apparent lack of this domain in each of other, more basal groups of fungi certainly disfavors this hypothesis. The authors should compare the sequence of OB-N to that of OB-1 and OB-2. One possibility is that OB-N in basiomycetes represents an independent acquisition of a fourth OB-fold either from an ancient duplication of one of the other OB-folds in Pot1 or perhaps even from some other protein. Comparison of OB-N to OB-1 and OB-2 could help address at least the former possibility. The authors should also discuss a bit more their search comparisons to other fungi. Have all available fungi in Ascomycota, Zoopagomycota and Chytridiomycota been examined? Finding even a single non-basiomycete with the OB-N could greatly strengthen the possibility of multiple independent loss events of OB-N in these other fungal groups. The authors might also want to mention the possibility that an independent acquisition of an OB-N in basiomycetes could conceivably be accompanied by some degree of convergent evolution that might lead to some resemblance to the metazoan OB-N.

The OB fold is defined minimally by the five beta strands, but the alpha helix separating strand 3 and 4 is quite common. We have provided a reference (Theobald *et al.,* Annu Rev Biophys Biomol Struct. 32:115-33) for this (page 5, line 130).

The structures displayed in the original Fig. 1C looked somewhat distinct because of different extent of modeling (i.e., different lengths of N- and C-terminal extensions were included in the model by the program based on similarities to the template). To better illustrate the similarity of the modeled structures

to OB folds, we now show a superposition of one of the predicted structures (*Pa*Pot1 OB-N) on a *bone fide* OB fold domain (human RPA70 OB-A) (Fig 1C and page 5, line 133-135).

The secondary structure prediction is generated by Promals by considering the structural propensities of all the sequences in the alignment, so there are no separate predictions for different OB folds. We have highlighted the sequence of the *Pa*Pot1 OB-N domain that is modeled in Fig. 1C.

As to the origin of basidiomycetes Pot1 OB-N, we have now compared this domain to OB1 and OB2 of basidiomycete Pot1 homologs (S2 Fig, part A and B). Neither of these OB domains exhibited stronger resemblance to basidiomycete OB-N than metazoan OB-N. We also evaluated the possibility that the basidiomycete Pot1 might have acquired OB-N from RPA70 (per reviewer 3). Again, phylogenetic analysis does not support this idea (S2 Fig, part C). To search for OB-N in other fungal lineages, we examined ~ 50 hits in Zoopagomycota and Chytridiomycota, representing close to all identifiable homologs in these basal branches. We were unable discover any Pot1 with a plausible OB-N. Similarly, we examined more than 100 Pot1 hits in Ascomycota and could not identify an OB-N. We have qualified our conclusion concerning Pot1 OB-N evolution and have mentioned the alternative possibility of independent acquisition followed by convergent evolution (page 5, line 147-149).

P5-6: Some details of the strand-exchange assay should be mentioned in the text. Minimally, the authors should describe whether the assay shown involves telomeric or non-telomeric sequences and what the sequences of the oligos used was. I don't know if there are technical problems with examining strand exchange with repetitive sequences but doing the reaction with telomeric and non-telomeric repeats would be very interesting. Oligos with either the ss or the ds region telomeric could be used. One might guess that Pot1 would be better at causing strand exchange with telomeric compared to non-telomeric sequences, especially since the reported effect in the data here is quite modest. Strand exchange that favored telomeric sequence would be both especially interesting and harder for skeptics to ignore.
The assays employed oligonucleotides described in a published paper, and these oligonucleotides do not harbor telomere repeats. This is now mentioned in the text (page 6, line 180-182). We agree that it would be interesting to study strand exchange using telomere-like substrates. However, to rigorously examine the effect of Pot1 on Rad51-mediated strand exchange on telomere DNA *in vitro*, one would ideally also include other relevant factors such as Brh2, Trf2, etc. We are interested in eventually pursuing this line of study, but it is beyond the scope of current work.

Define the asterisks in the legend to Fig. 2D.
The asterisks mark the positions of the P^{32} label. This is now indicated in the figure legend (page 27, line 872-873).

The authors should discuss the additional bands seen in the strand exchange assay, most notably in Fig. 2D and what evidence proves the particular band expected to be the result of strand exchange is actually that. An arrow pointing to this specific band would be helpful too. At least one of the other bands varies in intensity quite considerably, increasing when Pot1 is present.
The substrates and products in the reactions are now marked by arrowheads (Fig. 2C and 2D). There is one band just below the products that appears to be Pot1-dependent. While the identity of this is unclear, it is not Rad51-dependent and probably not relevant to strand exchange. This is now discussed in the figure legend (page 27, line 876-878).

P6: The variable telomere length phenotype of U. maydis wild type cells complicates trying to prove modest average length changes but the data presented seem adequate for arguing that the Pot1 mutation and the Rad51 mutation do indeed have somewhat shorter telomeres than wild type. The authors argue that the physical interactions of Pot1 and Rad51 and the apparently similar telomere shortening in pot1 delta N and rad51 mutants suggests that these proteins act in the same telomere maintenance pathway. However, the authors' data in Fig. 5 suggests that brh2 delta mutants also may have similarly shortened telomeres. This ought to be further examined. If true, this would add more information to help elucidate the connection between HR and telomeres in *U. maydis*.
Yes, the *brh2*Δ mutant exhibits comparable shortening of telomeres as *rad51*Δ. We have previously reported this as well as the telomere-association of Brh2 (Yu *et al.* DNA repair 12, 472.) This is now

described in the text and noted as providing support for the idea that *rad51* and *brh2* probably cooperate to facilitate telomere maintenance (just as they do in mammalian cells), and that *pot1* may regulate how this function is executed (page 9, line 269-273).

P7/Fig. 4B: It would be worthwhile to have quantification of the relative telomere signal in the mutants. While this would be subject to some issues regarding interpretation, it would still be interesting to estimate.
The quantitation is now included in Fig. 4B.

Fig. 5C: G circle and C circle data are stated as being shown but only C circle data is shown.
Corrected. Only C-circle analysis was performed (page 28, line 917-918).

Fig. 6B legend appears to refer to Fig. 6D.
The legends to Fig. 6B and 6D have been corrected (page 29, line 928 and line 934).

Reviewer #2:

Zahid et al. reported the functions of Ustilago maydis Pot1 in telomere maintenance and protection in this manuscript. The authors found that Ustilago Pot1 has an extra OB fold at its N-terminus, interacts with Rad51 and, interestingly, stimulates Rad51's strand exchange activity. Using the arabinose-responsive crg1 promoter, the authors were able to overexpress
POT1/pot1ΔN (when growing cells on YPA) or turn their expression off (when growing cells on YPD). The authors showed that Pot1 is essential for cell growth. Depletion of Pot1 leads to greater telomere length heterogeneity, an elevated telomeric C-circle level, increased amounts of single-stranded C and G-rich telomeric DNA, increased levels of both C and G strand TERRA, and more telomeric RNA:DNA hybrids. Many of these phenotypes are suppressed by rad51Δ and brh2Δ. The data support the conclusion that Pot1and Rad51 play critical roles in telomere maintenance and telomere recombination.

Concerns:

1. There is no estimation of crg1 driven POT1/pot1ΔN expression at the RNA or protein level when cells were grown on YPA and YPD compared to POT1 expression driven by its endogenous promoter. Some of the phenotypes, particularly for pol1ΔN growing on YPA, probably results from the overexpression of this mutant. In addition, if crg1 driven POT1/pot1ΔN expression can be depleted completely, the phenotypes for both POT1 and pot1ΔN cells grown on YPD is expected to be the same. The fact that they have very different phenotypes suggests that the depletion is not complete.

We agree with the reviewer's interpretation that the some of the phenotypes of the mutants are due to over-expression in YPA. We also agree that the depletion of Pot1 proteins in $pot1^{crg1}$ and $pot1\Delta N^{crg1}$ grown in YPD is probably incomplete. Indeed, we found that the level of *pot1* RNA in $pot1^{crg1}$ grown in YPA is about 10-fold higher than normal *pot1* RNA, whereas that in YPD is about 1/2 to 1/3 of normal pot1 level (S5 Fig, part E). These observations and interpretations are now discussed in more detail in the text (page 8, line 235-237, line 244-249).

2. As a relevant point, POT1 OB1/2 and OB3 both can interact with RAD51, albeit the affinity appears to be lower for OB3. Still, this could contribute to the phenotypes of *crg1* driven pot1ΔN grown on YPA. This is not discussed.
We have expanded the discussion of $pot1\Delta N^{crg1}$ grown in YPA to include this point as well as possibly altered interaction between Pot1ΔN and other DNA repair factors (page 8, line 244-249).

3. rad51Δ and brh2Δ suppressed the increased TERRA (both C and G strand) level in the pot1 mutant. This may be an effect on TERRA transcription, but it may also be due to increased TERRA degradation (or reduced TERRA stability). No evidence has been shown that the suppression is at the transcription level. Therefore, the statement on page 9 line 282 should be revised.

This statement has been revised to include the possibility of increased RNA stability (page 10, line 307-308).

Reviewer #3:

In this manuscript, Dr. Lue and his colleagues identified an extra N-terminal OB fold (OB-N) in fungal Ustilago maydis Pot1 (UmPot1). By sequence alignment, the authors showed that this OB-N is present in most metazoans. Using in vitro biochemical studies, the authors further demonstrated that UmPot1 OB-N, together with OB1 and OB2, binds directly to Rad51 and regulates its strand exchange activity. Deletion of OB-N domain alone resulted in telomere shortening, while deletion of Pot1 through transcriptional repression resulted in growth arrest and severe telomeric structural aberrations as well as high level of TERRA. In addition, the phenotypes can be largely suppressed by co-deletion of rad51 and brh2. Thus, the authors conclude that UmPot1 maintains telomere integrity under both normal and deprotected states through regulation of two major HDR repair proteins Rad51 and Brh2. Overall, the experiments were well designed and carefully characterized. The data is of high quality and presented in a clear and convincing manner. I have a few comments listed below:
1. The authors mentioned evolutionary relationships of RPA like complexes at telomeres. It would be interesting to check the sequence/2D/3D structural similarity between OB-N domain in UmPot1 and the OB fold in RPA1 or CTC1 to get an idea whether UmPot1 is a "chimeric" RPA-Pot1 form.
We have evaluated the possibility that the basidiomycete Pot1 OB-N might have been acquired from basidiomycetes RPA70 rather than having a common origin with metazoan Pot1 OB-N (S2 Fig, part C). However, phylogenetic comparison indicates that the basidiomycete Pot1 OB-N is more similar to metazoan OB-N than to RPA70 OB-N. Similar analysis were performed for the basidiomycete Pot1 OB1 and OB2 (per reviewer 1) with similar conclusions. We have not analyzed the OB folds of CTC1 for this purpose because recent structural analysis suggests that it is structurally quite different from Pot1 even in terms of domain organizations (Lim et al. Science 368:1081-5; Cai et al. bioRxiv. 2021:2021.12.17.473235.)

2. In Figure 3, TRF Southern blot showed Pot1ΔN strain had clear telomere shortening, while the deletion seems not affecting overall growth by serial dilution. Have the authors followed a long-term growth effect of pot1ΔN strain? Or in the presence of replication stress regents like HU to see whether it accelerates telomere shortening and senescence?
We have passaged *pot1ΔN* for 6 streaks (~150 generation) and have not observed evidence of senescence (Fig. 3B and page 7, line 191-192). We also analyzed the effect of HU on telomeres in the parental strain UCM350 and *pot1ΔN.* However, even low concentrations of HU caused enough replication stress to induce telomere recombination, and there is no strong difference between UCM350 and *pot1ΔN* (S4 Fig, part B and page 7, line 201-204).

3. The deletion of Pot1 (Pot1crg1 and Pot1ΔNcrg1) resulted in a substantial increase of ssDNA shown in Figure 4. Are those ssDNA generated by aberrant HDR or by resection, or are those ssDNA located internally or terminally? Is it possible to distinguish it by adding the Exo I digestion?
We have analyzed the sensitivity of the G- and C-strand ssDNA to the 3'-overhang specific Exo I and the 5' overhang specific $RecJ_f$. Interestingly, both the C-strand ssDNA (abundant in *pot1*^{crg1}) and the G-strand ssDNA (abundant in *pot1ΔN*^{crg1}) are partially sensitive to both nucleases (S5 Fig, part D). Because chromosome-end G- and C-overhangs should be sensitive only to Exonuclease I and $RecJ_f$, respectively, this result point to linear ECTRs as the source of much of the ssDNA (page 8, line 227-229). It seems likely that both resection and resolution of HDR intermediates could be involved in the production of linear ECTRs enriched for ssDNA, but a lot more studies would be required to understand the underlying mechanisms.

4. The OB-N is present in most basidiomycete, but not in fission yeast and other filamentous fungi. Have the authors considered generating a chimeric fusion Pot1 with the OB-N from UmPot1 fused with other fission yeast or filamentous fungi Pot1?
Given the limited knowledge we have concerning how OB-N interacts with and modulates Rad51 activity, it may be premature to test such fusion proteins. With more knowledge about the Pot1-interaction site on

Rad51 and its conservation in fission yeast and filamentous fungi, we would be in a better position to design a good fusion experiment for a 3-OB Pot1.

Minor points:
1. In Figure 1D, it will be easier for the readers to have the scheme of the truncation variants as shown in S2 figure a.
Included (Fig. 1D).

2. From the biochemical binding experiment, have the authors calculate the stoichiometry ratio of Pot1 and Rad51?
We have not attempted to determine the stoichiometry of binding because the amount of Rad51 that associates with Pot1 is quite low and most likely sub-saturating.
